# Mobile-Master-Vehicle-Based LiDAR Perception and Centralized Control of Sensor-Light Slave Vehicles

**DOI:** 10.3390/s26185983

**Published:** 2026-09-21

**Authors:** Heeseok Shin, Jeonghoon Kwak, Heechang Moon, Sangjun Bae, Nguyen Xuan Mung, Myeongjun Kim

**Affiliations:** 1 Convergence Major for Intelligent Drone, Department of Aerospace Systems Engineering Sejong University, Seoul 05006, Republic of Korea; hsshin9017@sejong.ac.kr (H.S.);; 2 AI+X Research Center, Advanced Institute of Convergence Technology (AICT), Suwon 16229, Republic of Korea; jeonghoon@snu.ac.kr; 3 Department of Mechanical and System Design Engineering, Hongik University, Seoul 04066, Republic of Korea; hcmoon@hongik.ac.kr; 4 Department of Drone Robotics, Sejong Cyber University, Seoul 05000, Republic of Korea; sj.bae@sjcu.ac.kr; 5 Department of Electrical and Control Engineering, Cheongju University, Cheongju 28503, Republic of Korea

**Keywords:** cooperative perception, external vehicle tracking, LiDAR-based localization, vehicle-model-aided tracking, master ego-motion compensation, centralized vehicle control, sensor-light vehicle, path tracking

## Abstract

This study presents an asymmetric cooperative perception-and-control architecture in which a sensor-rich mobile master vehicle performs external perception, state estimation, motion planning, and path-tracking control for sensor-light slave vehicles. The proposed vehicle-model-aided LiDAR tracking (VMALT) method combines ego-motion-compensated LiDAR measurements with transmitted speed and steering commands through a kinematic bicycle model. In physical-vehicle experiments, VMALT reduced the slave-position RMSE from 0.36 m with a LiDAR-only constant-velocity Kalman filter to 0.32 m, corresponding to an 11.1% improvement. The estimated state was subsequently used for closed-loop speed and path-tracking control of the physical slave vehicle. Scalability was evaluated using a one-master–two-slave configuration over three separate runs comprising circular and linear paths and geometrically identified line-of-sight-overlap candidates. Concurrent two-target LiDAR availability ranged from 96.465% to 99.934%, with a maximum interior observation gap of 0.10 s. The VMALT trajectories expressed in the GPS coordinate frame followed the position and direction of both slave vehicles, with heading RMSEs ranging from $1.835^{\circ }$ to $4.609^{\circ }$. In an LTE-tethering communication test, all 2400 UDP packets were returned, with median and 99th-percentile round-trip times of 4.654 and 8.220 ms, respectively. These results demonstrate the feasibility of centralized perception and control for multiple sensor-light vehicles under the evaluated low-speed operating conditions.

## 1. Introduction

Advances in artificial intelligence, sensor fusion, and real-time control have expanded autonomous driving beyond road transportation to logistics, industrial transportation, disaster response, and unmanned mobility. Conventional autonomous vehicles independently perform perception, localization, decision-making, path planning, and vehicle control using multiple onboard sensors and high-performance computing resources. Although this onboard-centered architecture provides a high degree of autonomy, equipping every vehicle with an independent perception and computing system increases cost, power consumption, and hardware complexity.

Cooperative perception addresses the limited sensing range and occlusion of individual vehicles by sharing raw sensor data, object-level information, or intermediate features through V2V and V2I connectivity [1,2,3,4,5]. Full-stack cooperative driving frameworks have also been developed to connect cooperative perception with planning and vehicle control [6,7]. However, most existing approaches assume that each participating vehicle has its own perception sensors and computing resources and fuses shared information with its local observations. Comparatively few studies have considered an asymmetric architecture in which a sensor-rich vehicle externally perceives a sensor-light vehicle and centrally generates its driving commands.

External LiDAR systems have been investigated for estimating vehicle position, velocity, and trajectory [8,9,10,11]. These studies primarily use stationary roadside infrastructure. When LiDAR is mounted on a moving vehicle, its measurements contain both sensor-vehicle ego-motion and target-vehicle motion, requiring relative-motion compensation. Recent cooperative-perception studies have also addressed heterogeneous feature fusion, real-world V2X evaluation, and communication-efficient information sharing [12,13,14,15,16]. Furthermore, temporal asynchrony, relative-pose errors, and object motion can degrade the spatial and temporal consistency of collaborative observations [17,18,19,20].

This study proposes an asymmetric cooperative perception-and-control architecture in which a mobile master vehicle performs external perception, state estimation, speed planning, and path-tracking control for sensor-light slave vehicles. The master vehicle serves as a mobile ground control station (GCS) equipped with 3D LiDAR, localization sensors, and high-performance computing resources. It externally estimates the position, velocity, and heading of the slave vehicle and generates target-speed and steering commands. The slave vehicle does not use an onboard perception system or high-level autonomous-driving computer; it executes the commands transmitted by the master vehicle.

The main contributions of this study are as follows:

- An asymmetric cooperative architecture is presented in which a mobile master vehicle integrates external perception, state estimation, and centralized control for sensor-light slave vehicles.
- VMALT is proposed by combining master ego-motion compensation, command-aware vehicle-model prediction, and LiDAR measurement updates.
- The proposed method is quantitatively evaluated against LiDAR-only CV-KF using independent RTK-GPS ground truth. A stronger LiDAR-only IMM-EKF baseline is additionally evaluated in complementary closed-loop simulation. Physical closed-loop control, one-master–two-slave operation, multi-target observation continuity, and LTE-tethering communication performance are evaluated under low-speed operating conditions.

## 2. Related Work

### 2.1. V2X-Based Cooperative Perception

Cooperative perception enables multiple sensing agents to share information through vehicle-to-vehicle (V2V) or vehicle-to-infrastructure (V2I) communication, thereby extending the limited field of view of individual vehicles and mitigating sensor occlusion. Depending on the type of shared information, cooperative perception methods are commonly categorized as early fusion of raw sensor data, intermediate fusion of learned features, or late fusion of object-level detection results [1].

Several datasets and benchmarks have accelerated the development of V2X cooperative perception. OPV2V provides a simulation dataset and an OpenCOOD-based benchmark for evaluating different fusion strategies in V2V environments [2]. V2X-Sim supports multi-agent, multimodal, and multitask perception involving both vehicles and roadside units [3], whereas DAIR-V2X provides real-world LiDAR and camera data for vehicle–infrastructure cooperative 3D object detection [4]. V2V4Real and TUMTraf-V2X further enable cooperative perception and tracking to be evaluated using real-world V2V and vehicle–infrastructure measurements [5,13].

Communication-efficient cooperative perception has also received considerable attention. DiscoNet learns a collaboration graph through knowledge distillation [14], whereas Where2comm selectively transmits spatially informative features using confidence maps [15]. How2comm combines spatial-channel filtering with mutual-information-based communication to balance perception performance and communication bandwidth [16]. V2X-ViT uses a transformer-based architecture to integrate heterogeneous multiscale features while accounting for asynchronous communication and pose uncertainty [12].

These studies primarily aim to improve object-detection coverage and accuracy by fusing information from multiple sensor-equipped agents. They generally assume that each participating vehicle has its own perception sensors and computing resources. In contrast, the architecture investigated in this study assigns external perception, state estimation, and control-command generation to a sensor-rich master vehicle, whereas the sensor-light slave vehicle executes the transmitted commands without performing independent high-level perception or decision-making.

### 2.2. External LiDAR-Based Vehicle Tracking

External LiDAR systems can estimate the positions, velocities, and trajectories of road users within their sensing range and have therefore been investigated as sensing components for cooperative driving automation [8]. A conventional geometric LiDAR tracking pipeline typically consists of region-of-interest filtering, ground or background removal, point-cloud clustering, object-position extraction, data association, and temporal state estimation.

Liu et al. extended the detection range of low-channel roadside LiDAR through static-background construction [9]. Lin et al. combined L-shape fitting, object classification, data association, and Kalman filtering to estimate vehicle trajectories from roadside LiDAR measurements [10]. A subsequent method combined ResNet18-based detection with an improved Hungarian algorithm to maintain tracking continuity during short-term occlusion [11].

Most external LiDAR tracking studies assume stationary roadside sensors. Because the sensor coordinate system remains fixed, the observed temporal displacement can be associated directly with target-vehicle motion. When LiDAR is mounted on a moving master vehicle, however, the observations contain both master-vehicle ego-motion and slave-vehicle motion. Estimating the global state of the slave vehicle therefore requires compensation for the time-varying position and orientation of the master vehicle. This moving-sensor configuration distinguishes the present study from conventional roadside-LiDAR tracking.

### 2.3. Motion Compensation and Cooperative Vehicle Control

Pose uncertainty and sensor asynchrony can cause spatial and temporal misalignment among collaborative observations. Latency-aware collaborative perception introduced SyncNet to temporally align asynchronous features [17]. CoAlign employs an agent–object pose graph and multiscale feature alignment to compensate for relative-pose errors [18]. FFNet predicts infrastructure-side temporal feature flow to compensate for asynchronous vehicle–infrastructure measurements [19], whereas MRCNet accounts jointly for pose noise, object motion, and perception uncertainty in robust cooperative communication [20].

These methods primarily improve spatial or temporal alignment at the feature level. The proposed vehicle-model-aided LiDAR tracking (VMALT) method instead operates directly at the state-estimation level. It combines ego-motion-compensated LiDAR positions with the speed and steering commands generated by the master vehicle and predicts the slave-vehicle state using a kinematic bicycle model. Each predicted state is subsequently corrected using a newly acquired LiDAR measurement. VMALT therefore uses command information already available to the centralized controller as an input to external vehicle tracking.

Previous studies have also extended cooperative perception to vehicle planning and control. OpenCDA provides a cooperative driving automation framework integrating perception, localization, planning, and control modules [6]. CooperNAUT connects LiDAR representations shared through V2V communication to an end-to-end driving policy [7]. However, these frameworks assume that each connected vehicle performs local sensing and perception before sharing information with other vehicles.

The present study addresses a different cooperative configuration in which perception and control authority are distributed asymmetrically. A sensor-rich mobile master vehicle performs external perception, slave-state estimation, speed planning, and path-tracking control, while the sensor-light slave vehicle applies the transmitted commands to its actuators. The main research gap addressed in this study is therefore the integration of moving-master LiDAR tracking, command-aware vehicle-model prediction, and centralized slave-vehicle control within a physical closed-loop system.

## 3. Methodology

### 3.1. Cooperative Perception-and-Control Architecture

The proposed architecture consists of a sensor-rich mobile master vehicle and a sensor-light slave vehicle. The master vehicle is equipped with a 3D LiDAR, a positioning sensor, and a high-performance computing unit. It externally perceives the slave vehicle, estimates its position, velocity, and heading, and generates speed and steering commands with respect to a predefined reference path. The slave vehicle does not perform high-level perception or autonomous-driving computation; it applies the commands received through an LTE-based communication link to its actuators.

**Figure 1:**
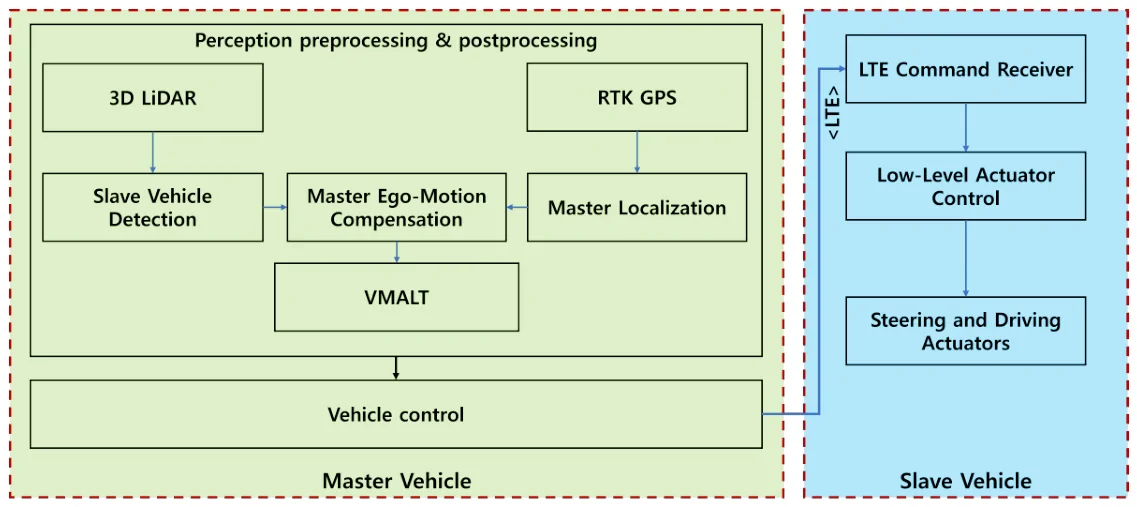
Proposed master–slave cooperative architecture integrating external LiDAR tracking, VMALT, and centralized control.

As shown in Figure 1, the master vehicle functions as a mobile ground control station (GCS). Its control commands are used both to actuate the slave vehicle and to predict the slave-vehicle motion in the proposed vehicle-model-aided LiDAR tracking (VMALT). This command feedback distinguishes the proposed method from conventional LiDAR-only tracking.

The system assumes planar and low-speed vehicle motion, negligible lateral tire slip, and a known slave-vehicle wheelbase. The RTK-GPS receiver installed on the slave vehicle is used only as independent ground truth and does not provide any input to the tracking or control algorithms.

The same asymmetric structure can support multiple slave vehicles by maintaining an independent track, VMALT state, and command channel for each slave on the master computer. For slave *i*, the master maintains $x_{S_{i},k}$ and generates $u_{i,k}={\left[v_{\mathrm{cmd},i,k},\delta_{\mathrm{cmd},i,k}\right]}^{T}$. This extension does not require a perception or high-level planning module on the slave vehicles. Its feasibility is evaluated using one master and two physical slave vehicles in Section 5.3.

### 3.2. External LiDAR Measurement and Ego-Motion Compensation

The master-vehicle LiDAR point cloud is processed using voxel-grid downsampling, RANSAC-based ground removal, and Euclidean clustering [21]. The slave-vehicle cluster is selected based on its geometric dimensions and proximity to the predicted track, and its centroid is used as the position measurement.

To compensate for master-vehicle ego-motion, the detected centroid is transformed from the LiDAR frame *L* to the global frame *W*:

(1) $$W_{{\bar{z}}_{S,k}}=W_{T_{M,k}}M_{T_{L}}L_{{\bar{c}}_{S,k}}$$

where $L_{{\bar{c}}_{S,k}}$ is the homogeneous cluster-centroid measurement in the LiDAR frame, and $W_{{\bar{z}}_{S,k}}$ is the corresponding slave-vehicle position measurement in the global frame. The matrices $M_{T_{L}}$ and $W_{T_{M,k}}$ transform the measurement from the LiDAR frame to the master-vehicle frame and from the master-vehicle frame to the global frame, respectively. The master pose is interpolated at each LiDAR timestamp, and the resulting global measurement is used identically by LiDAR-only CV-KF and VMALT.

### 3.3. Vehicle-Model-Aided LiDAR Tracking

#### 3.3.1. LiDAR-Only Tracking Baseline

The baseline employs a constant-velocity Kalman filter (CV-KF) with the state $x_{k}^{\mathrm{CV}}={\left[p_{S,k}^{T},v_{S,k}^{T}\right]}^{T}$, where $p_{S,k}$ and $v_{S,k}$ denote the global position and velocity vectors of the slave vehicle at the *k*th LiDAR measurement, respectively. Here, *S* denotes the slave vehicle, *k* denotes the measurement index, and the superscript *T* denotes matrix transpose.

The state is predicted under the constant-velocity assumption and corrected using the ego-motion-compensated LiDAR centroid. The planar speed and heading are calculated as

(2) $${\hat{v}}_{S,k}=\sqrt{{\hat{v}}_{x,S,k}^{2}+{\hat{v}}_{y,S,k}^{2}}\;\;{\hat{\psi }}_{S,k}=\mathrm{atan2}\left({\hat{v}}_{y,S,k},{\hat{v}}_{x,S,k}\right)$$

where ${\hat{v}}_{x,S,k}$ and ${\hat{v}}_{y,S,k}$ are the estimated velocity components of the slave vehicle along the global *x*- and *y*-axes, respectively. Their resultant magnitude ${\hat{v}}_{S,k}$ represents the planar speed, and ${\hat{\psi }}_{S,k}$ represents the heading calculated from the direction of the velocity vector.

#### 3.3.2. Command-Aware VMALT

VMALT incorporates the transmitted speed and steering commands into the slave-vehicle state prediction. The state and input vectors are defined as $x_{S,k}^{\mathrm{VM}}={\left[x_{S,k},y_{S,k},\psi_{S,k}\right]}^{T}$ and $u_{k}={\left[v_{\mathrm{cmd},k},\delta_{\mathrm{cmd},k}\right]}^{T}$, respectively.

Here, $\left(x_{S,k},y_{S,k}\right)$ and $\psi_{S,k}$ denote the global position and heading of the slave vehicle, while $v_{\mathrm{cmd},k}$ and $\delta_{\mathrm{cmd},k}$ denote the speed and steering commands transmitted to the slave vehicle.

The slave-vehicle state is predicted using a kinematic bicycle model:

(3) $$\begin{array}{cc} x_{S,k+1} & =x_{S,k}+v_{\mathrm{cmd},k}\cos\psi_{S,k}\Delta t_{k},\\ y_{S,k+1} & =y_{S,k}+v_{\mathrm{cmd},k}\sin\psi_{S,k}\Delta t_{k},\\ \psi_{S,k+1} & =\mathrm{wrap}\left(\psi_{S,k}+\frac{v_{\mathrm{cmd},k}}{L_{S}}\tan\delta_{\mathrm{cmd},k}\Delta t_{k}\right). \end{array}$$

Here, $\Delta t_{k}$ is the sampling interval and $L_{S}$ is the slave-vehicle wheelbase. The function wrap(·) normalizes the heading angle to (−π,π].

An extended Kalman filter corrects the predicted position using the planar components of the ego-motion-compensated LiDAR measurement $W_{{\bar{z}}_{S,k}}$ defined in Equation (1). When no valid LiDAR measurement is available, VMALT retains the vehicle-model prediction.

### 3.4. Centralized Speed Planning and Path-Tracking Control

The slave-vehicle state estimated by VMALT is used for centralized speed planning and path-tracking control. The reference speed is determined from the forward path curvature $\kappa_{k}$:

(4) $$v_{\mathrm{ref},k}=\mathrm{sat}\left(\frac{v_{\max}}{\left(\right|\kappa_{k}\left|+\epsilon \right)k_{\kappa }},v_{\min},v_{\max}\right),$$

where $v_{\min}$ and $v_{\max}$ are the minimum and maximum reference speeds, $k_{\kappa }$ is the curvature-dependent speed gain, and ϵ is a small positive constant that prevents division by zero. The function $\mathrm{sat}(a,a_{\min},a_{\max})$ limits *a* to the interval $\left[a_{\min},a_{\max}\right]$.

A speed-dependent look-ahead distance is used for Pure Pursuit control:

(5) $$L_{d,k}=\mathrm{sat}\left(k_{s}v_{\mathrm{ref},k},L_{d,\min},L_{d,\max}\right),$$

where $k_{s}$ is the speed-dependent gain, and $L_{d,\min}$ and $L_{d,\max}$ are the minimum and maximum look-ahead distances, respectively.

Let $\alpha_{k}$ denote the angle between the estimated slave-vehicle heading and the direction from the estimated vehicle position to the selected look-ahead point. The unconstrained steering command is

(6) $$\delta_{\mathrm{cmd},k}^{*}=\arctan\left(\frac{2L_{S}\sin\alpha_{k}}{L_{d,k}}\right),$$

where $L_{S}$ is the slave-vehicle wheelbase. The superscript ∗ denotes the steering command before applying the steering-angle and steering-rate constraints.

The constrained speed and steering commands are transmitted to the slave vehicle and used as inputs for the subsequent VMALT prediction. This establishes a command-aware closed loop between state estimation and centralized control.

## 4. Experimental Setup

The physical-vehicle validation was conducted in two stages. The first stage used a one-master–one-slave configuration to evaluate the state- estimation performance of VMALT against LiDAR-only CV-KF and to verify the complete closed-loop chain from external perception to physical vehicle control. The second stage extended the architecture to a one- master–two-slave configuration and evaluated simultaneous multi-target observation, independent slave control, and path driving under different relative vehicle arrangements. Communication latency, jitter, and response timeout were additionally measured over the LTE-tethering network used by the vehicle computers. The RTK-GPS measurements mounted on the slave vehicles were used for evaluation and were not provided to the online tracking or control algorithms.

### 4.1. Experimental Platform

**Figure 2:**
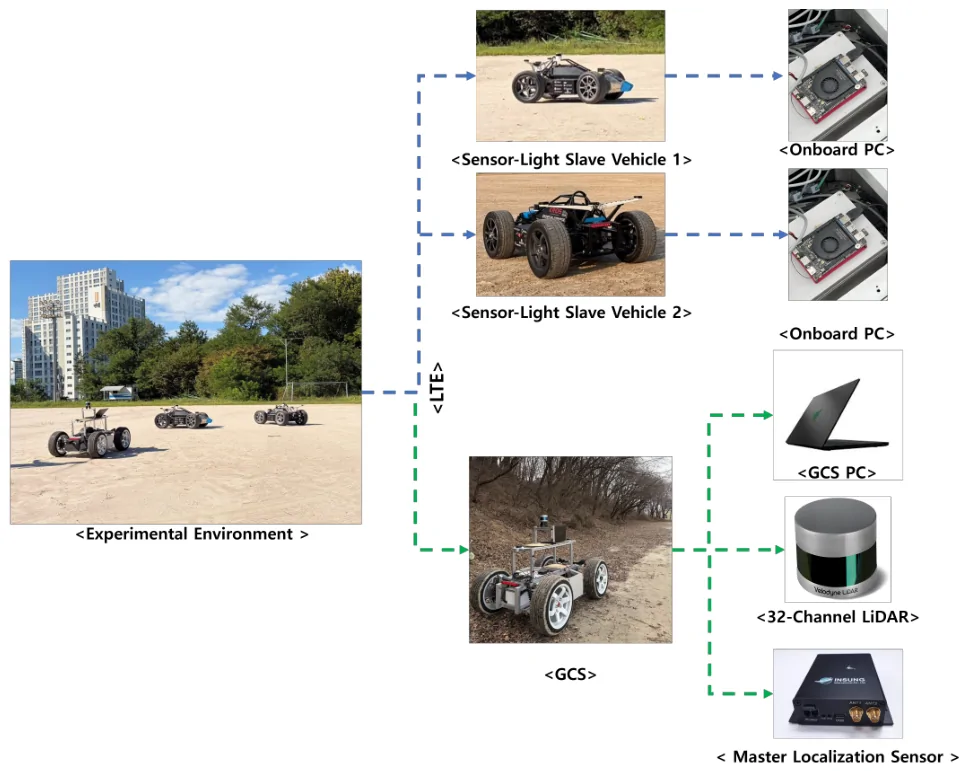
Physical vehicles used in the two-stage validation: one sensor-rich master vehicle and two sensor-light slave vehicles. The one-master–one-slave configuration was used for the initial VMALT and closed-loop validation, and the one-master–two-slave configuration was used for the extended multi-vehicle evaluation.

The experimental platform consisted of one sensor-rich mobile master vehicle and two sensor-light slave vehicles, as shown in Figure 2. The master vehicle was equipped with a 32-channel LiDAR, a positioning sensor, and a high-performance computing unit. It performed point-cloud processing, master ego-motion compensation, slave-state estimation, speed planning, and path-tracking control. The first-stage experiments used the master and one slave, whereas the second-stage experiments used the master and both slaves.

Each slave vehicle was equipped with a LattePanda IOTA for receiving the speed and steering commands generated by the master vehicle and applying them to its actuators. The slave-side computers did not perform environmental perception, path planning, or high-level path-tracking control.

The hardware configuration of the master and slave vehicles is summarized in Table 1. The speed and steering commands were transmitted through an LTE-based communication link between the vehicles. To characterize this link in the same network configuration, two Windows computers were connected to one LTE-tethering network. A client transmitted 256-byte UDP packets at 20 Hz for 120 s, and the server immediately echoed each received packet. Sequence numbers and monotonic timestamps were recorded to calculate application-level round-trip time (RTT), adjacent-RTT jitter, response timeout, deadline misses, duplication, and packet reordering. The parameters used for VMALT and centralized control are summarized in Table 2.

### 4.2. Experimental Procedure

During the state-estimation experiment, the master vehicle traveled southward along a straight path at a constant speed of 1.0 m/s while observing the slave vehicle with its LiDAR. The LiDAR point cloud was acquired at approximately 10 Hz. The master pose was interpolated at each LiDAR timestamp and used to transform the detected slave-vehicle position into the global coordinate system.

LiDAR-only CV-KF and VMALT used the same point-cloud preprocessing, slave-cluster detection, and ego-motion-compensated LiDAR measurements. Therefore, the comparison isolated the effect of the tracking prediction model. LiDAR-only CV-KF used a constant-velocity model, whereas VMALT used the transmitted speed and steering commands with the kinematic bicycle model.

The RTK-GPS installed on the slave vehicle measured its global position, velocity, and heading. These measurements were synchronized with the LiDAR timestamps only during post-processing. Position and velocity measurements were linearly interpolated, whereas heading was interpolated after angle unwrapping.

In the subsequent closed-loop experiment, only VMALT was used to provide the feedback state to the master-side controller. The master vehicle generated speed and steering commands from the predefined reference path, transmitted them through the LTE-based link, and controlled the physical slave vehicle. This experiment evaluated the integrated operation of external perception, state estimation, command generation, communication, and slave-side actuation.

These state-estimation and closed-loop experiments constituted the first validation stage and used one master and one slave. After confirming the operation of VMALT and the complete control loop in this configuration, the second stage introduced an additional slave vehicle to evaluate the extension of the architecture to simultaneous two-slave operation.

For the multi-slave evaluation, the master vehicle observed two physical slave vehicles simultaneously. The relative trajectories, range, bearing, and fitted dimensions of both targets were recorded at approximately 10 Hz in three independent runs. Two RTK-GPS receivers recorded the absolute trajectories and speeds of the slave vehicles. C1 and C2 denote the first and second circular-route conditions, respectively, whereas L1 denotes the first linear-route condition with a change in the lateral ordering of the two slaves. These conditions varied the path shape, inter-vehicle geometry, and observation bearing. The evaluation included intervals in which the target bearings approached each other, producing geometrically challenging partial line-of-sight overlap.

The reference paths for the two slave vehicles were defined from GPS coordinates. During closed-loop operation, the master GPS provided the master pose, while the master LiDAR provided the relative observations of both slaves. VMALT combined the ego-motion-compensated LiDAR observations with the transmitted motion commands to estimate both slave states, which were used by the master-side controller to drive the two vehicles along the GPS-based paths. The RTK-GPS receivers mounted on the slave vehicles independently recorded the resulting trajectories for evaluation.

To express the logged VMALT track positions and RTK-GPS measurements in a common coordinate system, each LiDAR target was associated with one slave vehicle and the LiDAR timestamps were converted to GPS-week time. No spatial scale factor was estimated. For C2 and L1, the synchronized positions of the two VMALT tracks and the two RTK-GPS receivers were used to calculate a frame-wise planar rigid transformation consisting only of rotation and translation. The projected centroid trajectories were smoothed before their tangential directions were calculated, and heading was expressed clockwise from UTM north, consistently with the RTK-GPS convention.

For the complementary closed-loop baseline simulation, CV-KF, IMM-EKF, and VMALT were independently connected to the same Pure Pursuit controller. The LiDAR-only IMM-EKF combined constant-heading/velocity and coordinated-turn EKF modes without using the transmitted control commands. Three path geometries and three vehicle-response conditions were evaluated using 30 paired random-noise trials per combination.

For the communication evaluation, 2400 UDP requests were scheduled at 20 Hz. An echo was classified as a timeout when no response was received within 3 s, and a round-trip deadline miss was recorded when RTT exceeded 100 ms. Jitter was evaluated from the absolute RTT difference between adjacent successfully returned sequence numbers.

### 4.3. Evaluation Metrics

The tracking performance was evaluated using planar position, velocity, and heading RMSE. Let ${\hat{p}}_{S,k}$ and $p_{S,k}^{\mathrm{GT}}$ denote the estimated and RTK-GPS-based planar positions of the slave vehicle, respectively. The position RMSE is defined as

(7) $${\mathrm{RMSE}}_{p}=\sqrt{\frac{1}{N}\sum_{k=1}^{N}{∥{\hat{p}}_{S,k}-p_{S,k}^{\mathrm{GT}}∥}_{2}^{2}}$$

The velocity RMSE is calculated as

(8) $${\mathrm{RMSE}}_{v}=\sqrt{\frac{1}{N}\sum_{k=1}^{N}{\left({\hat{v}}_{S,k}-v_{S,k}^{\mathrm{GT}}\right)}^{2}}$$

The heading RMSE accounts for angular periodicity:

(9) $${\mathrm{RMSE}}_{\psi }=\sqrt{\frac{1}{N}\sum_{k=1}^{N}{\mathrm{wrap}}^{2}\left({\hat{\psi }}_{S,k}-\psi_{S,k}^{\mathrm{GT}}\right)}$$

Closed-loop longitudinal performance was evaluated using the difference between the reference speed generated by the master vehicle and the measured speed of the slave vehicle:

(10) $${\mathrm{RMSE}}_{\mathrm{speed}}=\sqrt{\frac{1}{N}\sum_{k=1}^{N}{\left(v_{\mathrm{ref},k}-v_{\mathrm{act},k}\right)}^{2}}$$

Path-tracking performance was evaluated in the controller domain. For the VMALT-estimated position ${\hat{p}}_{S,k}$ and reference path $P_{\mathrm{ref}}$, the path error is defined as the minimum Euclidean distance to the reference path:

(11) $$e_{\mathrm{path},k}=\min_{p\in P_{\mathrm{ref}}}{∥{\hat{p}}_{S,k}-p∥}_{2}$$

The controller-domain path RMSE is calculated as

(12) $${\mathrm{RMSE}}_{\mathrm{path}}=\sqrt{\frac{1}{N}\sum_{k=1}^{N}e_{\mathrm{path},k}^{2}}$$

Because this metric is calculated from the VMALT-estimated position used by the controller, it represents the internal closed-loop consistency between the estimated trajectory and the reference path.

For two-target LiDAR observation, let $d_{i,k}\in \left\{0,1\right\}$ indicate whether target *i* has a recorded output in frame *k*. The per-target availability and concurrent two-target availability are

(13) $$A_{i}=\frac{1}{N}\sum_{k=1}^{N}d_{i,k},\;A_{\mathrm{conc}}=\frac{1}{N}\sum_{k=1}^{N}d_{1,k}d_{2,k}.$$

Stable concurrent availability was additionally calculated over the common interval in which both targets had established tracks. The line-of-sight-overlap candidate flag was determined from the target bearing separation and the angular half-width derived from the fitted target dimensions. This flag identifies challenging geometry and is used to analyze observation continuity.

For the coordinate-registration analysis, the planar residual was the Euclidean distance between a GPS-anchored projected VMALT track and the corresponding RTK-GPS position. Heading error was calculated using the wrapped angular difference between the projected trajectory tangent and the RTK-GPS heading.

For communication sample *k*, the application-level RTT is denoted by $r_{k}$. Adjacent-RTT jitter and echo-timeout percentage are defined as

(14) $$j_{k}=\left|r_{k}-r_{k-1}\right|,\;P_{\mathrm{timeout}}=100\frac{N_{\mathrm{sent}}-N_{\mathrm{echo}}}{N_{\mathrm{sent}}}.$$

The RTT distribution is summarized using its mean, median, 95th and 99th percentiles, and maximum. Because RTT is measured with the client monotonic clock, it includes both communication directions and the application processing time.

## 5. Results and Discussion

### 5.1. Slave-Vehicle State-Estimation Results

**Figure 3:**
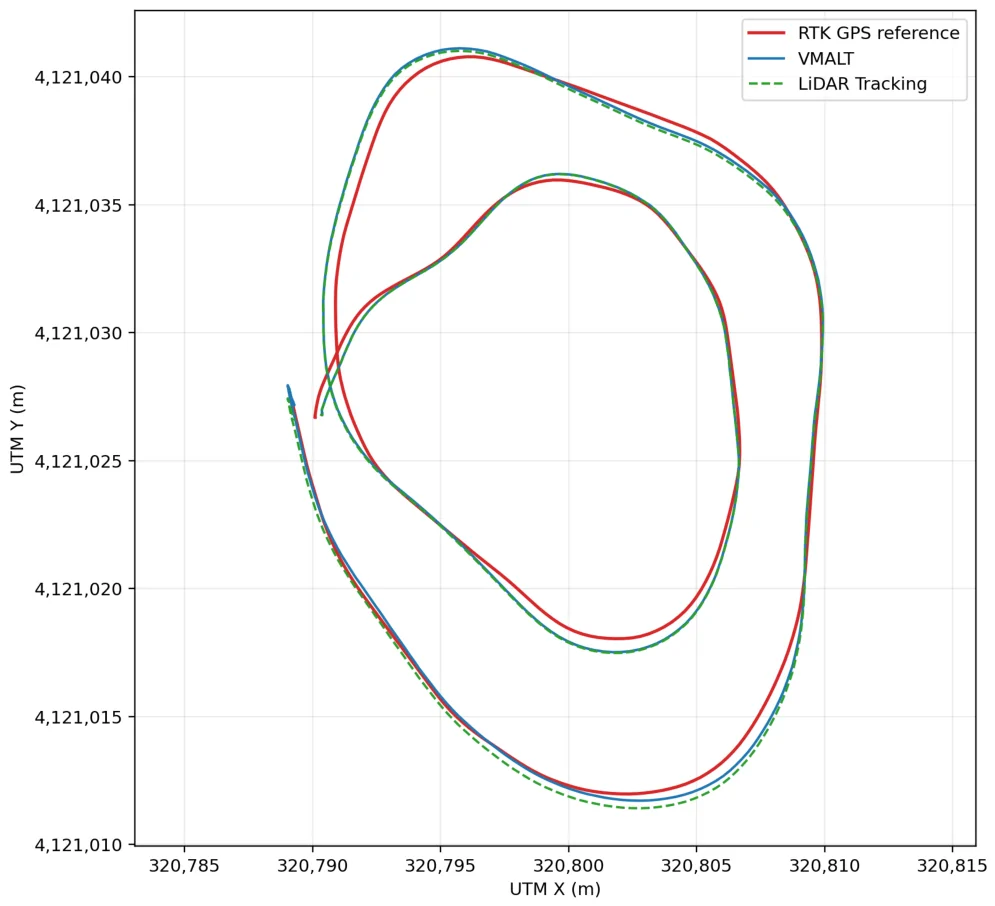
Slave-vehicle trajectories obtained from RTK-GPS, LiDAR-only CV-KF, and VMALT.

Table 3 summarizes the state-estimation performance relative to the RTK-GPS ground truth. Figure 3 compares the RTK-GPS ground-truth trajectory with the trajectories estimated by LiDAR-only CV-KF and VMALT. Both methods reproduced the overall motion of the slave vehicle. However, the LiDAR-only CV-KF trajectory exhibited a systematic southward offset from the RTK-GPS trajectory. The direction of this offset coincided with the southward motion of the master vehicle, which traveled at 1.0 m/s while observing and controlling the slave vehicle.

Although the nominal master ego-motion was removed by transforming the LiDAR centroid into the global frame, the CV-KF prediction relied only on the previously estimated position and velocity. It therefore remained sensitive to frame-to-frame centroid variations associated with changes in relative viewing geometry and LiDAR point distribution. The alignment between the offset direction and master motion, together with the reduced offset obtained after command-aware prediction was introduced under the same measurement preprocessing, is consistent with this explanation. However, the present data do not independently identify the contribution of each possible source of centroid bias; the mechanism is therefore presented as an evidence-supported interpretation rather than a direct causal measurement.

In contrast, the VMALT-estimated trajectory showed a smaller southward offset and closer agreement with the RTK-GPS trajectory. VMALT did not apply an artificial spatial correction to the LiDAR measurements. Instead, it predicted the subsequent slave-vehicle state using the transmitted speed and steering commands together with the kinematic vehicle model. This command-aware prediction constrained the estimated motion to remain consistent with the feasible speed and direction of the slave vehicle, reducing the influence of centroid variations that were inconsistent with the predicted vehicle motion.

The position RMSE decreased from 0.36 m for LiDAR-only CV-KF to 0.32 m for VMALT, corresponding to an 11.1% reduction. Under the tested moving-master condition, this result is consistent with the command-aware vehicle-model prediction reducing the influence of directional centroid variation on the estimated position.

**Figure 4:**
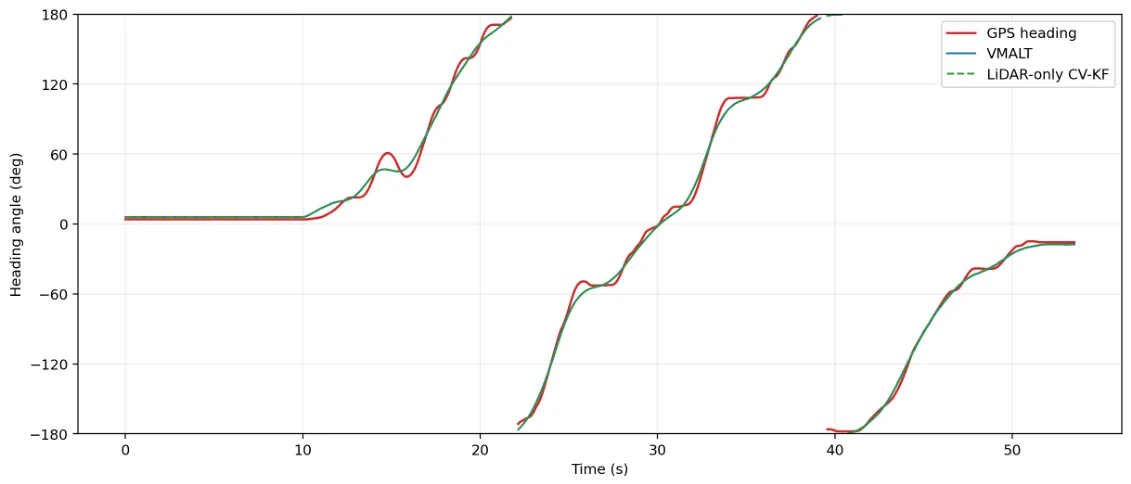
Slave-vehicle heading estimates from LiDAR-only CV-KF and VMALT compared with RTK-GPS measurements.

Figure 4 shows that both methods followed the overall heading variation, although frame-to-frame fluctuations remained. In LiDAR-only CV-KF, the heading was calculated from the estimated velocity vector; therefore, small variations in the velocity components resulted in relatively large heading fluctuations, particularly at low speeds.

**Figure 5:**
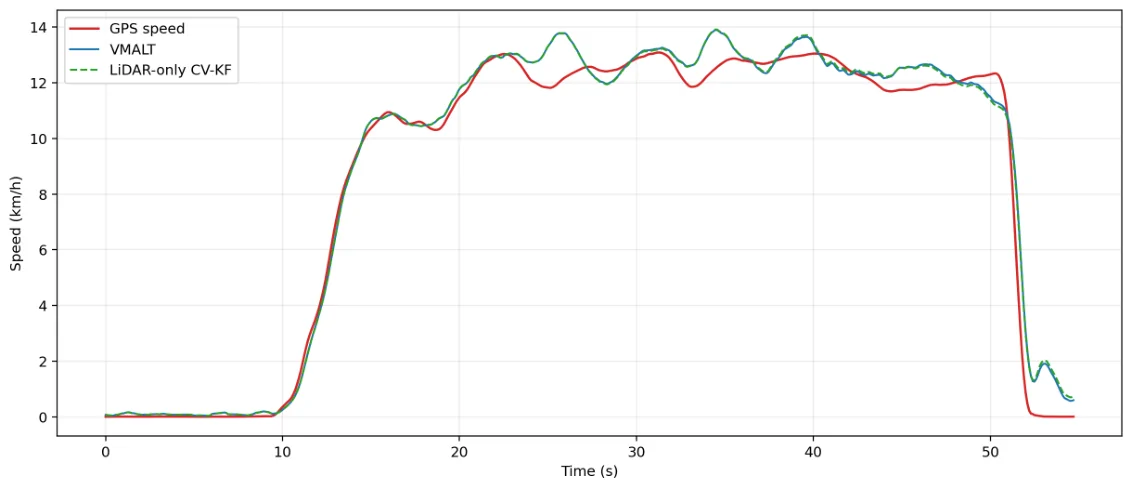
Slave-vehicle velocity estimated using LiDAR-only CV-KF and VMALT compared with RTK-GPS.

Figure 5 shows that both methods captured the overall velocity variation of the slave vehicle. The LiDAR-only CV-KF exhibited local fluctuations because its velocity estimate depends on temporal changes in the detected cluster centroid. Consequently, small frame-to-frame centroid variations can be amplified when converted into velocity.

The heading RMSEs of LiDAR-only CV-KF and VMALT were $4.178^{\circ }$ and $4.183^{\circ }$, respectively. The difference of $0.005^{\circ }$ is negligible, indicating comparable heading-estimation performance under the tested conditions. Thus, the primary quantitative benefit of VMALT was observed in position estimation rather than heading estimation.

VMALT used the transmitted speed and steering commands directly as the inputs of the kinematic bicycle prediction in Equation (3). The kinematic bicycle model describes planar motion geometry; a first-order actuator-response state was not included in the estimator. The gradual response mentioned here refers only to the observed physical vehicle behavior after a command change and explains why discrepancies remained during speed transitions. This distinction between the estimator motion model and the physical actuator response has been made explicit to avoid treating them as two alternative vehicle models.

The velocity RMSEs of LiDAR-only CV-KF and VMALT were 0.181 and 0.179 m/s, respectively. Their similar overall values are attributable to the relatively low-speed driving condition and the continuous availability of LiDAR measurements, under which the constant-velocity assumption already provided a reliable baseline. VMALT therefore maintained comparable velocity-estimation accuracy while providing a more motion-constrained estimate.

### 5.2. Closed-Loop Speed- and Path-Tracking Results

Following the state-estimation experiment, VMALT was integrated into the real-time external perception and control system of the master vehicle. The VMALT-estimated position, speed, and heading were used as feedback for curvature-based speed planning and Pure Pursuit path-tracking control. The resulting speed and steering commands were transmitted through the LTE-based communication link and applied to the actuators of the physical slave vehicle. LiDAR-only CV-KF was not evaluated as a separate physical-vehicle closed-loop controller. A controlled simulation-based closed-loop comparison is reported in Section 5.5 as complementary model-level evidence.

Table 4 summarizes the closed-loop control performance.

**Figure 6:**
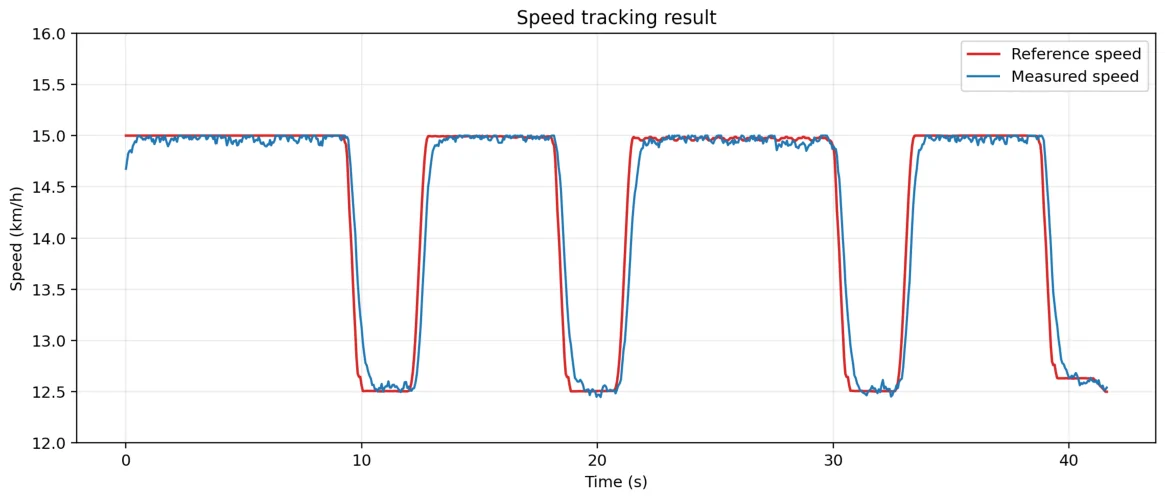
Closed-loop speed-tracking response of the physical slave vehicle, including the initial transient and subsequent tracking region.

Figure 6 compares the reference speed generated by the curvature-based speed planner with the measured speed of the physical slave vehicle. The largest difference occurred during the initial acceleration because the reference command was applied immediately, whereas the physical vehicle required a finite response time to reach the commanded speed. This initial difference produced the maximum speed-tracking error of 0.575 m/s.

After the initial transient, the measured speed converged toward the reference profile and followed its subsequent variations. Local differences appeared when the reference speed changed because the physical vehicle responded progressively to the updated command. These differences decreased after each speed transition rather than remaining as a sustained offset. Consequently, the speed-tracking RMSE was limited to 0.141 m/s, while the mean error was 0.066 m/s. The difference between the maximum error and the overall RMSE indicates that the largest error was concentrated in a short transient interval.

**Figure 7:**
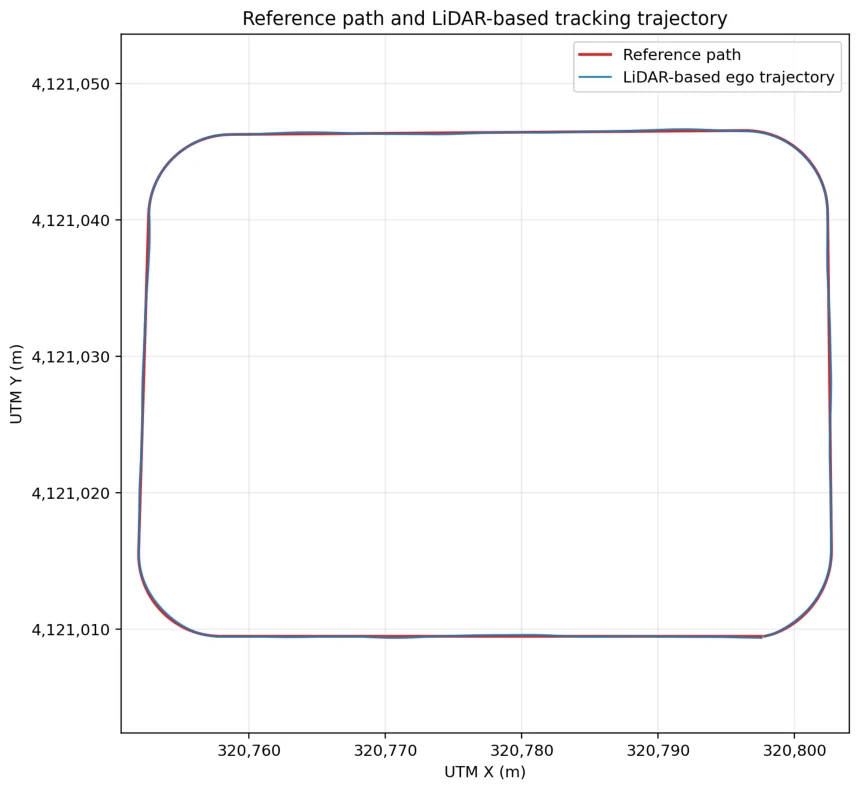
Closed-loop path-tracking result using the VMALT-estimated slave-vehicle state.

Figure 7 compares the reference path with the trajectory estimated by VMALT during the closed-loop experiment. The two trajectories closely overlapped along the straight and low-curvature sections. Local deviations increased near the curved sections, where the required steering angle and heading change were larger. This behavior is consistent with the speed-dependent look-ahead mechanism of Pure Pursuit, which generates smooth steering commands while progressively reducing the path error.

As the vehicle completed each curvature transition, the estimated trajectory converged toward the reference path. The resulting controller-domain path RMSE was 0.061 m, indicating that the VMALT-estimated state supported consistent path-tracking control over the experimental route.

Overall, the speed- and path-tracking results demonstrate the integrated operation of master-side external perception, VMALT-based state estimation, centralized command generation, LTE-based command transmission, and physical slave-vehicle actuation. The speed error was primarily concentrated in the initial transient, while the estimated trajectory remained close to the reference path throughout the closed-loop experiment.

### 5.3. One-Master–Two-Slave Experimental Results

**Figure 8:**
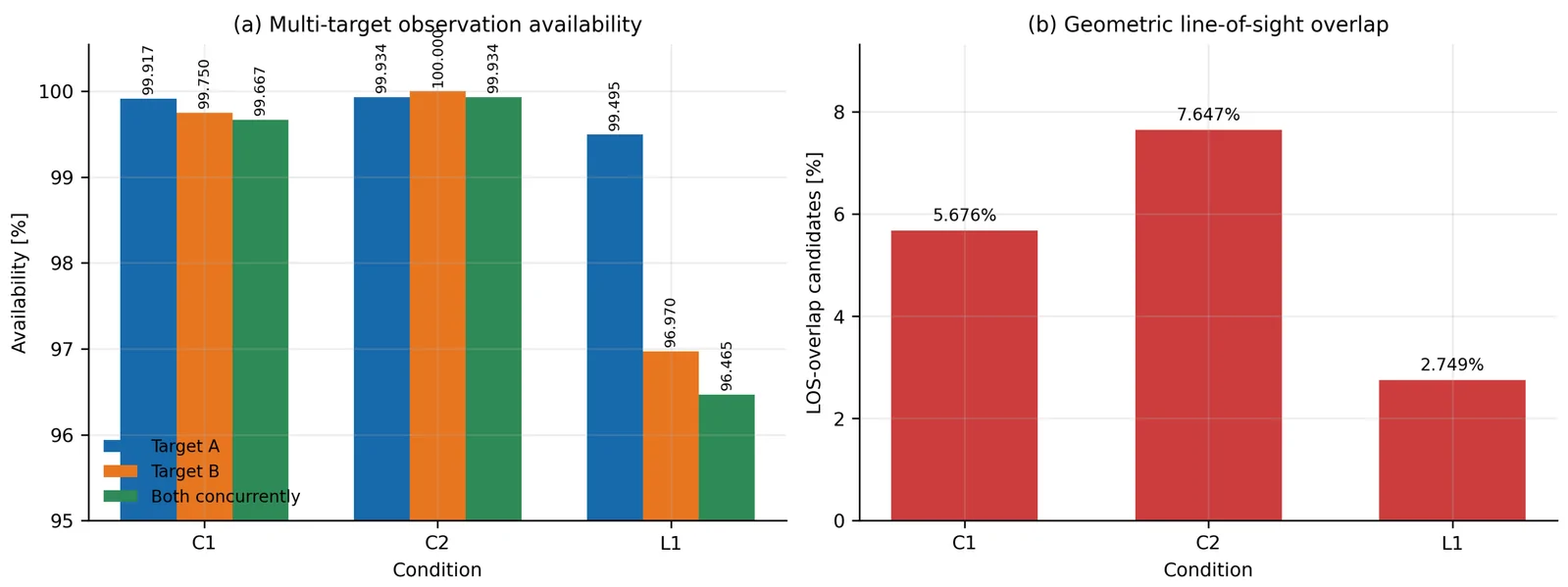
Multi-target LiDAR observation results: (**a**) per-target and concurrent two-target availability and (**b**) the proportion of geometric line-of-sight-overlap candidate frames in C1, C2, and L1.

**Figure 9:**
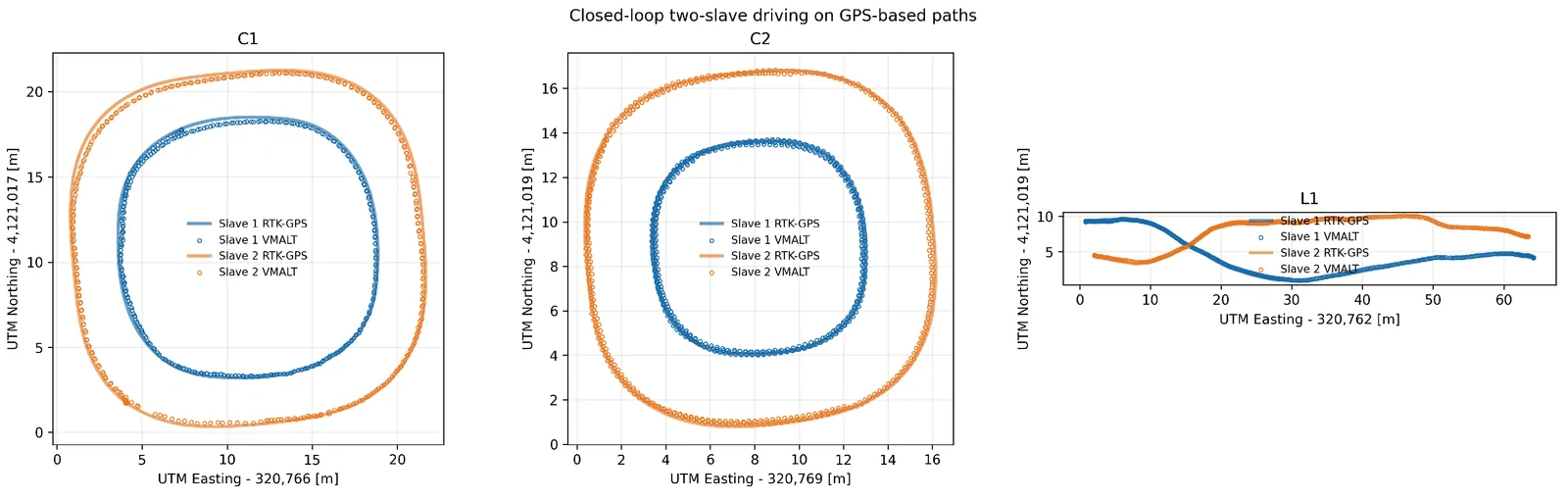
Closed-loop driving trajectories of two slave vehicles along GPS-based paths in C1, C2, and L1. The master combined its GPS pose and LiDAR observations through VMALT to estimate and control both slaves. Solid lines denote the trajectories recorded by the slave-mounted RTK-GPS receivers, and markers denote the corresponding VMALT tracks expressed in the same UTM frame.

The multi-slave experiment evaluated whether one sensor-rich master could observe and support two sensor-light slave vehicles under changes in path shape, inter-vehicle spacing, range, and viewing geometry. The experimental configuration and visibility conditions, together with the absolute RTK-GPS and projected VMALT trajectories for C1, C2, and L1, are presented in Figure 8 and Figure 9.

Table 5 summarizes the recorded controller-domain tracking-error signal and RTK-GPS speed. In C1, the tracking-error RMSEs of Slaves 1 and 2 were 0.589 and 0.543 m, respectively. In C2, the corresponding RMSEs were 0.364 and 0.435 m. Slave 2 therefore showed a 7.8% lower RMSE in C1 and a 19.6% higher RMSE in C2, indicating no consistent degradation for the second slave across the evaluated conditions. The mean absolute inter-slave speed differences were 0.185 km/h in C1 and 0.225 km/h in C2.

C1 had a larger mean inter-slave separation than C2 (12.413 m versus 8.952 m). Nevertheless, concurrent two-target availability remained 99.667% in C1, only 0.267 percentage points below the 99.934% obtained in C2, and the longest interior observation gap was 0.10 s in both conditions. The farther target in C1 was observed at a mean LiDAR range of 11.183 m and maintained 99.750% availability. Thus, the increased spacing in C1 did not produce a practically significant loss of multi-target observation continuity within the evaluated range.

Figure 8 summarizes per-target and concurrent availability and the proportion of line-of-sight-overlap candidate frames in C1, C2, and L1.

The concurrent two-target LiDAR observation results are summarized in Table 6.

Across the three runs, full-run concurrent availability ranged from 96.465% to 99.934%, while stable-interval concurrent availability ranged from 99.479% to 99.934%. The longest interior observation gap was at most 0.10 s. Geometric line-of-sight-overlap candidates occupied 2.749–7.647% of frames containing both targets. These results show that the master maintained high two-target observation continuity when the two slave vehicles changed their relative distance and bearing, including the visibility-challenging overlap geometry.

Figure 9 presents the closed-loop paths of the two slave vehicles. The reference paths were generated in the GPS coordinate frame, and the master drove both slaves by combining its GPS pose with LiDAR-based relative observations through VMALT. The solid lines are the trajectories independently recorded by the slave-mounted RTK-GPS receivers, while the markers are the corresponding master-side VMALT tracks expressed in the same UTM frame. The VMALT trajectories follow the position and direction of both slave vehicles in C1, C2, and L1.

**Figure 10:**
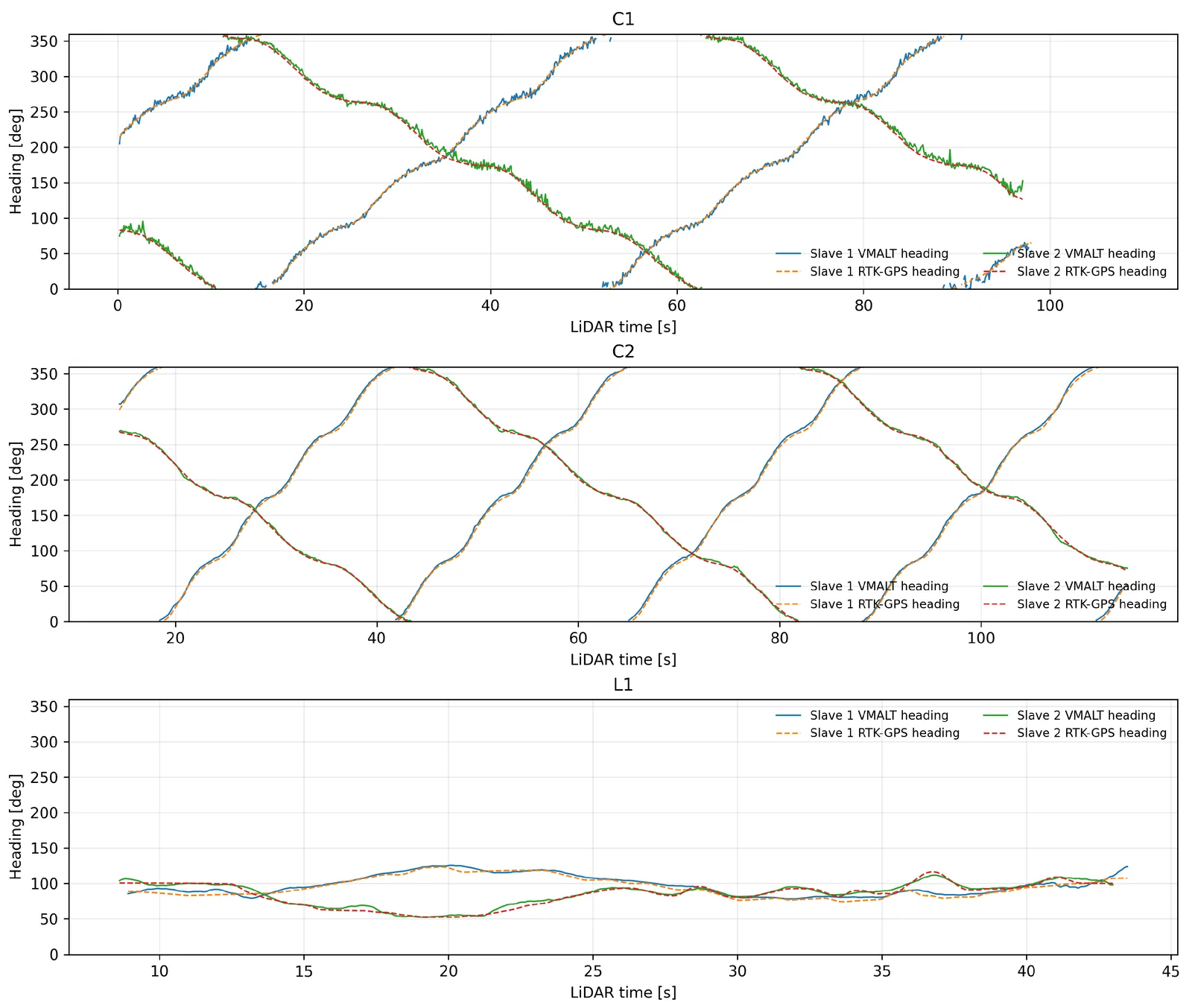
Heading comparison between the projected VMALT-track trajectory and the corresponding RTK-GPS measurement. Heading is defined clockwise from UTM north.

Figure 10 compares the direction of motion obtained from each projected VMALT trajectory with the corresponding RTK-GPS heading. Table 7 summarizes the coordinate-registration and heading-consistency results.

After expressing the VMALT estimates and the measured RTK-GPS values in the same UTM coordinate frame, their position and heading values were compared sample by sample. The resulting position RMSE ranged from 0.114 to 0.196 m, while the heading RMSE ranged from $1.835^{\circ }$ to $4.609^{\circ }$. The results show that the VMALT positions and directions were consistent with the corresponding RTK-GPS measurements over the evaluated C1, C2, and L1 conditions.

### 5.4. LTE Communication Performance

**Figure 11:**
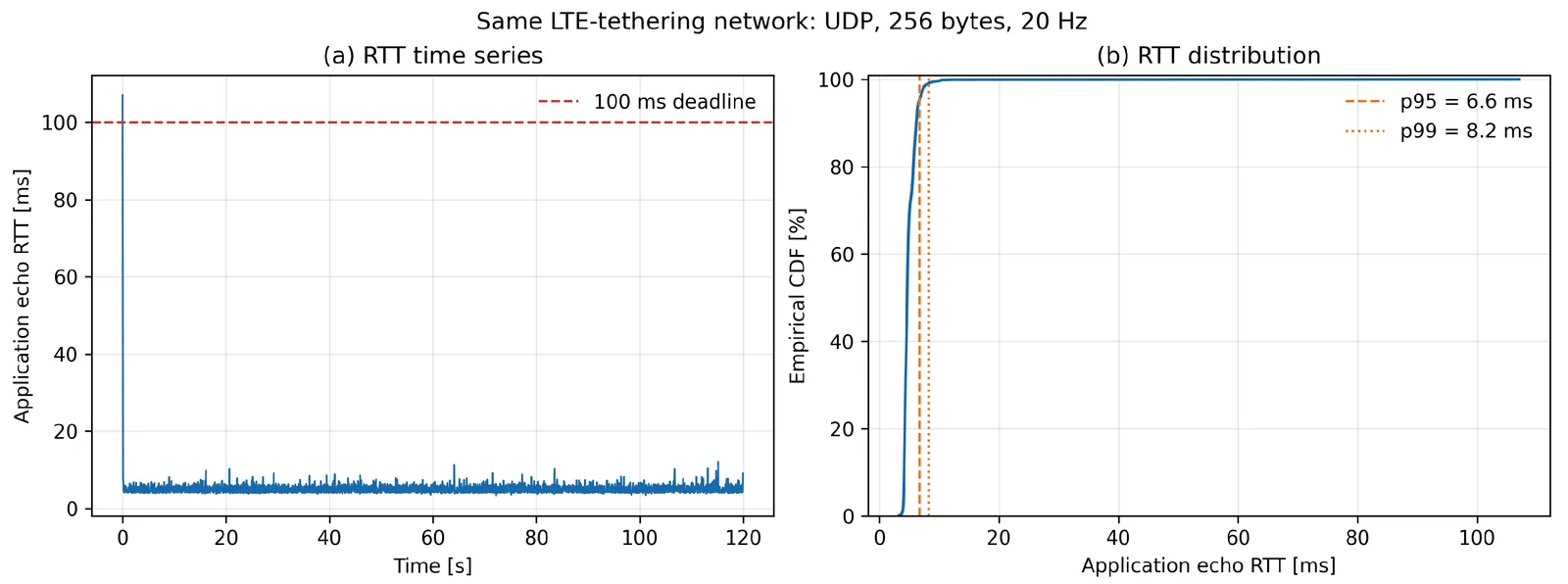
Application-echo RTT measured on the LTE-tethering network: (**a**) RTT time series with the 100 ms round-trip deadline and (**b**) empirical RTT distribution with the 95th and 99th percentiles.

Figure 11 shows the application-echo RTT time series and empirical cumulative distribution. All 2400 scheduled packets received a response. The mean, median, 95th-percentile, and 99th-percentile RTTs were 5.004, 4.654, 6.635, and 8.220 ms, respectively, and the maximum RTT was 107.051 ms. Mean adjacent-RTT jitter was 1.013 ms, with 95th- and 99th-percentile values of 2.504 and 4.196 ms. No echo timeout, duplication, reordering, or send error was observed. One packet exceeded the 100 ms round-trip deadline, corresponding to 0.042% of the transmitted packets.

The application-level LTE-tethering communication performance is summarized in Table 8.

The physical slave vehicles traveled at approximately 4.8–4.9 km/h. Using 5 km/h as a conservative reference, the vehicle travels only approximately 9.2 mm during the 95th-percentile RTT and 11.4 mm during the 99th-percentile RTT. Moreover, 2398 of 2400 packets (99.917%) were returned within the 50 ms command period corresponding to 20 Hz, and all packets were ultimately returned. Thus, under the evaluated low-speed and network conditions, communication delay and response loss were not large enough to interrupt continuous command delivery. This communication result is consistent with the recorded tracking-error RMSEs maintained by both slave vehicles in C1 and C2.

### 5.5. Simulation-Based Closed-Loop Baseline Comparison

A complementary closed-loop simulation compared CV-KF, IMM-EKF, and VMALT. Each estimator independently supplied pose feedback to the same Pure Pursuit controller. The LiDAR-only IMM-EKF combined a constant-heading/velocity EKF and a coordinated-turn EKF through probabilistic mode interaction. Unlike VMALT, neither CV-KF nor IMM-EKF used the transmitted speed or steering commands.

The evaluation used straight, S-curve, and lane-change paths and three plant conditions: an ideal response, a lagged actuator response, and a model-mismatch case with larger response time constants and an 8% wheelbase mismatch. Thirty paired random-noise trials were performed for each of the nine path–plant combinations. Paired trials used the same measurement-noise realization, controller parameters, and plant condition. Because each estimator independently supplied controller feedback, the resulting vehicle trajectories evolved independently. Path error was calculated from the simulated ground-truth vehicle position rather than from the estimated controller state.

**Figure 12:**
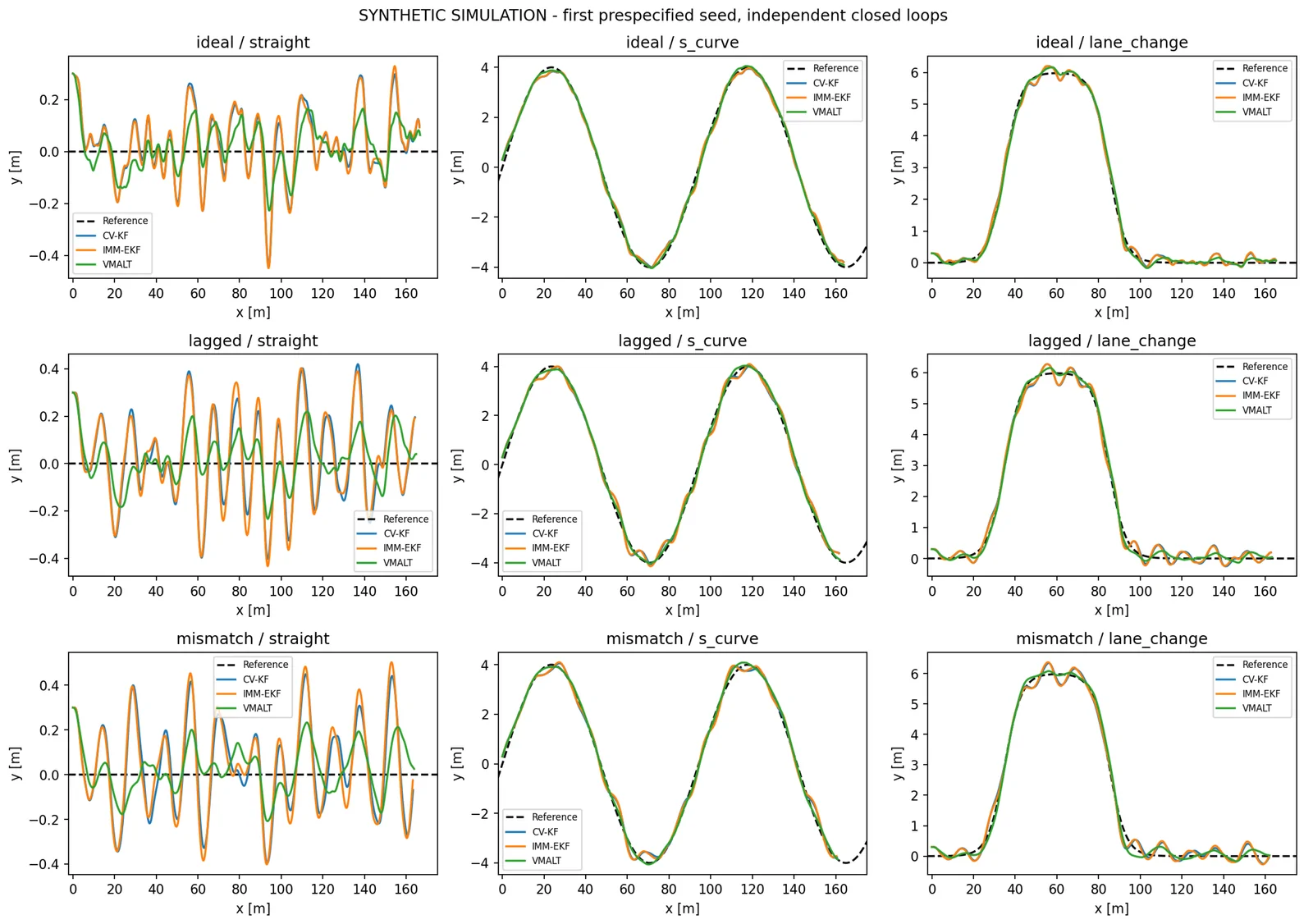
Simulation-based closed-loop comparison of CV-KF, IMM-EKF, and VMALT for straight, S-curve, and lane-change paths under ideal, actuator-lag, and model-mismatch vehicle conditions. Each panel shows the first pre-specified noise seed, whereas Table 9 summarizes the results from 30 paired trials per condition.

The simulation-based closed-loop comparisons of CV-KF, IMM-EKF, and VMALT are presented in Figure 12, while the corresponding ground-truth path RMSE statistics from 30 paired trials per condition are summarized in Table 9.

IMM-EKF produced comparable results to CV-KF under the ideal and actuator-lag conditions and achieved lower mean RMSE than CV-KF in all three model-mismatch conditions. It therefore provided a more competitive nonlinear, maneuver-aware baseline without using the transmitted control commands. VMALT achieved the lowest mean ground-truth path RMSE in all nine conditions. Compared with IMM-EKF, VMALT reduced the mean path RMSE by 0.0517–0.1087 m, corresponding to relative reductions of 35.7–48.4%. The unadjusted paired bootstrap 95% confidence intervals for the VMALT-minus-IMM-EKF differences did not include zero in any of the nine conditions.

These results provide complementary model-level evidence that the command-aware vehicle-model prediction improves closed-loop path tracking beyond both a linear constant-velocity filter and a command-free interacting multiple-model estimator. The simulation used assumed measurement-noise and actuator parameters that were not calibrated from the physical vehicles.

## 6. Conclusions

This study presented an asymmetric cooperative perception-and-control architecture in which a sensor-rich mobile master vehicle performs external perception, state estimation, motion planning, and centralized path-tracking control for sensor-light slave vehicles. The slave vehicles execute the transmitted speed and steering commands without requiring environmental perception, path planning, or high-level autonomous-driving computation.

VMALT combines master ego-motion compensation, LiDAR measurements, transmitted control commands, and a kinematic bicycle model. In the one-master–one-slave physical-vehicle experiment, VMALT reduced the RTK-GPS-based position RMSE from 0.36 to 0.32 m relative to LiDAR-only CV-KF. The velocity and heading RMSEs of the two methods were comparable, indicating that the primary improvement observed under the tested condition was in position estimation. When VMALT was integrated with the centralized controller, the physical slave vehicle achieved a speed-tracking RMSE of 0.141 m/s and a controller-domain path RMSE of 0.061 m. The latter value represents the consistency between the VMALT-estimated trajectory and the reference path and is distinct from an independently measured absolute path error.

The architecture was subsequently extended to a one-master–two-slave configuration. Over three runs comprising circular and linear paths, full-run concurrent two-target LiDAR availability ranged from 96.465% to 99.934%, stable-interval availability ranged from 99.479% to 99.934%, and the longest interior observation gap was 0.10 s. After the VMALT estimates and the measured RTK-GPS values were expressed in the same UTM frame, the sample-wise comparison produced position RMSEs of 0.114–0.196 m and heading RMSEs of 1.835–$4.609^{\circ }$.

In the LTE-tethering communication test, all 2400 UDP packets were returned. The median and 99th-percentile RTTs were 4.654 and 8.220 ms, respectively, and the mean adjacent-RTT jitter was 1.013 ms. At the evaluated vehicle speed, 99.917% of the packets were returned within the 50 ms command period. These results indicate that the measured communication delay and response loss did not interrupt continuous command delivery under the tested low-speed and network conditions.

A complementary closed-loop simulation compared CV-KF, IMM-EKF, and VMALT over three path geometries and three vehicle-response conditions. VMALT achieved a lower mean ground-truth path RMSE in all nine simulated conditions. Overall, the physical experiments demonstrate the feasibility of extending the proposed asymmetric architecture from one to two sensor-light slave vehicles. Future work will consider additional followers, denser traffic, prolonged target occlusion, and communication conditions beyond the evaluated LTE tethering network.

## Figures and Tables

**Table 1:** Hardware configuration of the experimental vehicles.

Component	Slave Vehicles	Master Vehicle
Computing platform	LattePanda IOTA	Razer Blade 14
Processor	Intel N150	AMD Ryzen 9 7940HS
Memory	LPDDR5, 8/16 GB	DDR5, up to 32 GB
GPU	Integrated graphics	NVIDIA GeForce RTX 40-series
Perception sensor	None	32-channel LiDAR
Positioning sensor	RTK-GPS for ground truth	Position and heading sensor
Primary function	Command execution	Perception, tracking, planning, and control

**Table 2:** Parameters used for VMALT and centralized control.

Parameter	Description	Value
$v_{\min}$	Minimum reference speed	0.0 m/s
$v_{\max}$	Maximum reference speed	4.17 m/s
$L_{d,\min}$	Minimum look-ahead distance	5 m
$L_{d,\max}$	Maximum look-ahead distance	10 m
$k_{s}$	Speed-dependent look-ahead gain	1.0
$k_{\kappa }$	Curvature-dependent speed gain	1.2
ϵ	Small positive constant	0.001
$L_{S}$	Slave-vehicle wheelbase	1100 mm

**Table 3:** State-estimation performance relative to RTK-GPS ground truth.

Method	Position RMSE (m)	Velocity RMSE (m/s)	Heading RMSE (°)
LiDAR-only CV-KF	0.36	0.181	4.178
Proposed VMALT	0.32	0.179	4.183

**Table 4:** Closed-loop speed- and path-tracking performance using VMALT.

Metric	Value
Mean speed-tracking error	0.066 m/s
Maximum speed-tracking error	0.575 m/s
Speed-tracking RMSE	0.141 m/s
Controller-domain path RMSE	0.061 m

**Table 5:** Two-slave speed and recorded controller-domain tracking-error results.

Condition	Vehicle	Speed (km/h)	Tracking RMSE (m)
C1	Slave 1	4.816±0.485	0.589
C1	Slave 2	4.863±0.154	0.543
C2	Slave 1	4.868±0.228	0.364
C2	Slave 2	4.863±0.150	0.435

**Table 6:** Concurrent two-target LiDAR observation performance.

Condition	Frames	Full Run (%)	Stable Interval (%)	Longest Gap (s)
C1	1202	99.667	99.667	0.10
C2	1518	99.934	99.934	0.10
L1	792	96.465	99.479	0.10

**Table 7:** Position and heading RMSEs between the VMALT estimates and the corresponding RTK-GPS measurements after projection into a common UTM coordinate frame.

Condition	Vehicle	Position RMSE (m)	Heading RMSE (°)
C1	Slave 1	0.161	3.716
C1	Slave 2	0.196	4.609
C2	Slave 1	0.124	3.112
C2	Slave 2	0.124	1.835
L1	Slave 1	0.114	3.758
L1	Slave 2	0.114	2.956

**Table 8:** Application-level LTE-tethering communication performance.

Metric	Value
Packets sent/echoed	2400/2400
RTT mean/median	5.004/4.654 ms
RTT 95th/99th percentile	6.635/8.220 ms
Maximum RTT	107.051 ms
Mean adjacent-RTT jitter	1.013 ms
Echo timeout	0.000%
100 ms deadline miss	0.042%

**Table 9:** Simulation-based ground-truth path RMSE over 30 paired trials per condition (mean ± standard deviation).

Plant	Path	CV-KF (m)	IMM-EKF (m)	VMALT (m)
Ideal	Straight	0.1392±0.0163	0.1401±0.0173	0.0884±0.0078
Ideal	S-curve	0.1508±0.0183	0.1483±0.0184	0.0893±0.0079
Ideal	Lane change	0.1525±0.0154	0.1517±0.0165	0.0975±0.0096
Lagged	Straight	0.1898±0.0323	0.1920±0.0330	0.1069±0.0108
Lagged	S-curve	0.1973±0.0286	0.1968±0.0309	0.1057±0.0106
Lagged	Lane change	0.1984±0.0293	0.1978±0.0304	0.1089±0.0121
Mismatch	Straight	0.2340±0.0531	0.2244±0.0512	0.1157±0.0127
Mismatch	S-curve	0.2352±0.0417	0.2238±0.0390	0.1176±0.0128
Mismatch	Lane change	0.2393±0.0541	0.2272±0.0520	0.1399±0.0163

## Data Availability

The data supporting the findings of this study are available from the corresponding author upon reasonable request.
